# Metabolic Control and Frequency of Clinical Monitoring Among Canadian Children With Phenylalanine Hydroxylase Deficiency: A Retrospective Cohort Study

**DOI:** 10.1002/jmd2.70042

**Published:** 2025-09-01

**Authors:** Nataliya Yuskiv, Ammar Saad, Beth K. Potter, Sylvia Stockler‐Ipsiroglu, John J. Mitchell, Steven Hawken, Kylie Tingley, Michael Pugliese, Monica Lamoureux, Andrea J. Chow, Jonathan B. Kronick, Kumanan Wilson, Annette Feigenbaum, Sharan Goobie, Michal Inbar‐Feigenberg, Julian Little, Saadet Mercimek‐Andrews, Amy Pender, Chitra Prasad, Andreas Schulze, Yannis Trakadis, Gloria Ho, Hilary Vallance, Valerie Austin, Anthony Vandersteen, Andrea C. Yu, Cheryl Rockman‐Greenberg, Aizeddin A. Mhanni, Pranesh Chakraborty

**Affiliations:** ^1^ Division of Biochemical Genetics, BC Children's Hospital, Department of Pediatrics University of British Columbia Vancouver British Columbia Canada; ^2^ School of Epidemiology and Public Health University of Ottawa Ottawa Ontario Canada; ^3^ Division of Medical Genetics Montreal Children's Hospital Montreal Canada; ^4^ Methodological and Implementation Research Program Ottawa Hospital Research Institute Ottawa Ontario Canada; ^5^ ICES uOttawa Ottawa Hospital Research Institute Ottawa Ontario Canada; ^6^ The Children's Hospital of Eastern Ontario Research Institute Ottawa Ontario Canada; ^7^ Division of Clinical and Metabolic Genetics, The Hospital for Sick Children University of Toronto Toronto Ontario Canada; ^8^ Department of Medicine University of Ottawa Ottawa Ontario Canada; ^9^ Bruyère Health Research Institute Ottawa Ontario Canada; ^10^ Ottawa Hospital Research Institute Ottawa Ontario Canada; ^11^ Division of Genetics Rady Children's Hospital and the University of California San Diego California USA; ^12^ Department of Pediatrics, Maritime Medical Genetics Service, IWK Health Dalhousie University Halifax Nova Scotia Canada; ^13^ Department of Medical Genetics, Faculty of Medicine & Dentistry University of Alberta Edmonton Alberta Canada; ^14^ Genetics & Metabolics McMaster Children's Hospital, Hamilton Health Sciences Hamilton Ontario Canada; ^15^ Department of Pediatrics (Section of Genetics and Metabolism) Western University London Health Sciences Centre London Canada; ^16^ Department of Paediatrics and Department of Biochemistry University of Toronto Toronto Ontario Canada; ^17^ The Hospital for Sick Children Toronto Ontario Canada; ^18^ Division of Medical Genetics, Department of Specialized Medicine McGill University Health Center Montreal Canada; ^19^ Department of Pathology and Lab Medicine BC Children's Hospital Vancouver British Columbia Canada; ^20^ Department of Pediatrics Children's Hospital of Eastern Ontario Ottawa Ontario Canada; ^21^ Department of Pediatrics and Child Health, Max Rady College of Medicine University of Manitoba Winnipeg Manitoba Canada; ^22^ Department of Pediatrics University of Ottawa Ottawa Ontario Canada

**Keywords:** inborn errors of metabolism, inherited metabolic disease, metabolic control, phenylketonuria

## Abstract

Achieving and maintaining metabolic control is critical for children with phenylalanine hydroxylase (PAH) deficiency. This retrospective longitudinal cohort study investigated metabolic control and monitoring frequency of children with PAH deficiency (≤ 12 years) treated at one of 12 pediatric metabolic centres across Canada. We abstracted data from medical charts and analyzed outcomes by age and diagnostic classification, using mixed effects regression. Of 215 children included in the study, 43% had a chart diagnosis of classic phenylketonuria (PKU); the remainder had a diagnosis of mild PKU or mild hyperphenylalaninemia (grouped as “less severe PAH deficiency”). During the first month of life, blood phenylalanine levels of children with classic PKU reached the target therapeutic range of 120–360 μmol/L at a median age of 15 days, but 74.3% and 32.9% had ≥ 1 and ≥ 3 values below 120 μmol/L, respectively. From age > 1 month to 12 years, mean blood phenylalanine values were 260.6 and 236.7 μmol/L for children with classic PKU and less severe PAH deficiency, respectively, with a trend of increased blood phenylalanine levels with increasing age (*p* < 0.001). Fewer children with classic PKU (37.2%) versus less severe PAH deficiency (77.9%) had > 60% of values in the therapeutic range, indicating less optimal metabolic control. Frequency of blood phenylalanine testing and communication with metabolic centres decreased with age. Our findings suggest a need to better understand the reasons for blood phenylalanine variability across child age and disease severity in order to inform supports for children with PAH deficiency and their caregivers to maintain metabolic control.

**Summary**

- We find that, among children across Canada age < 12 years and diagnosed with phenylalanine hydroxylase deficiency, blood phenylalanine levels are not always maintained within the recommended therapeutic range for metabolic control and suggest blood phenylalanine variability across child age and disease severity.

## 1 Introduction

Phenylketonuria (PKU) (OMIM #261600) is an inherited metabolic disease (IMD) characterized by a deficiency of phenylalanine hydroxylase (PAH) enzyme (OMIM #612349), highly expressed in the liver. This deficiency leads to impaired conversion of the essential amino acid phenylalanine to tyrosine and subsequent accumulation of phenylalanine in body fluids and tissues [1, 2]. PAH deficiency can cause mild to marked elevations of phenylalanine in body fluids and tissues [1, 3], which can be associated with severe and irreversible intellectual disability and other adverse outcomes if not treated and monitored vigilantly from early infancy [4, 5, 6, 7]. The diagnostic classification of PAH deficiency by metabolic phenotype varies widely across clinical settings due to differences in diagnostic methods [8, 9, 10, 11], including measuring blood phenylalanine levels [1, 12] and molecular genetic testing of pathogenic variants in the PAH gene [3].

A phenylalanine blood level measurement is included in the screening of all newborns in Canada. Following newborn screening, a diagnosis is confirmed by further laboratory testing [13]. In patients with confirmed PAH deficiency, immediate treatment consists of phenylalanine‐restricted diet therapy and, in some cases, pharmacological therapy along with ongoing close monitoring of blood phenylalanine levels in order to reach and maintain metabolic control [4, 5, 6, 7, 13, 14, 15, 16]. Guidelines for the medical and nutritional management of PAH deficiency recommend early initiation of treatment, preferably by the first week of life, targets for metabolic control by age, and monitoring frequency [5, 6, 7, 13, 16]. Across guidelines, there is a consensus on the importance of maintaining the majority of blood phenylalanine levels within a target therapeutic range across the lifespan to ensure metabolic control [7]. In Canada, this target range is 120–360 μmol/L, and evidence suggests poor long‐term outcomes when children's blood phenylalanine values remain outside this target range [17, 18]. For example, a meta‐analysis found that every 100 μmol/L increase in blood phenylalanine levels predicted a 1.3 to 3.1‐point reduction in intelligence quotient (IQ) scores among children with classic PKU, especially during the critical childhood period of 0–12 years of age [18]. While evidence on the impact of exposure to blood phenylalanine levels below the therapeutic range (i.e., < 120 μmol/L) is limited, there is a need to explore the frequency that such low values occur, especially during the first month of life when dietary modifications are being introduced.

Examining treatment practices and indicators of disease management for children with PAH deficiency is important for understanding how such guidance is translated into care and outcomes. To our knowledge, there is limited evidence on the state of metabolic control among Canadian children with PAH deficiency and the level of alignment between clinical recommendations and practice. The current study aimed to investigate metabolic control and the frequency of clinical monitoring of metabolic control among young children (aged 12 years or younger) with PAH deficiency treated at metabolic centres in Canada.

## 2 Methods

### 2.1 Study Design

We analyzed data collected in a retrospective longitudinal cohort study conducted by the Canadian Inherited Metabolic Disease Research Network (CIMDRN) [19]. Twelve metabolic genetic treatment centers in Canada that provide care to children with PKU participated in the CIMDRN cohort study and contributed data to this analysis.

### 2.2 Study Participants and Recruitment

Eligible children were: (a) born between 2006 and 2015; (b) received a diagnosis of PAH deficiency; and (c) received care at a participating metabolic centre. Children with hyperphenylalaninemia not due to PAH deficiency were excluded from the study. Clinicians identified eligible participants at their centres. Children were invited to participate in the study by staff members at participating centres. Parents provided consent and children assented when applicable [19].

### 2.3 Data Collection

Data were retrospectively abstracted from each participating child's medical record by designated research staff members at participating centres and entered into a standardized clinical data collection tool on Research Electronic Data Capture (REDCap), hosted at the Children's Hospital of Eastern Ontario [20, 21]. The period of data collection spanned from the child's birth until March 31 2017, or discharge from a participating centre, whichever came first [19].

We abstracted the following demographic data: child's sex assigned at birth, year of birth, enrolling treatment centre, and presence of siblings with PKU. Clinical and laboratory data abstracted were the child's chart‐documented metabolic phenotype group (i.e., diagnostic classification), newborn screening status, whether they received pharmacotherapy (sapropterin) to manage their PAH deficiency, as well as measured blood phenylalanine levels (i.e., dried blood spot and plasma samples) and the date of measurements. Abstracted data on monitoring frequency included the frequency of blood phenylalanine testing and the frequency of clinical interactions, defined as any in‐person or virtual communication between the family and the child's provider(s) at the metabolic centre.

#### 2.3.1 Diagnostic Classification of PAH Deficiency

We used the diagnostic classification documented in the child's medical chart to ascertain their PAH deficiency diagnosis: Classic PKU, mild PKU, or mild hyperphenylalaninemia. For analytic purposes, we grouped children with mild PKU or mild hyperphenylalaninemia as having “less severe PAH deficiency”. For children in the sample with a chart‐based diagnosis of classic PKU, we verified the diagnostic classification by investigating whether they had any recorded blood phenylalanine levels above 1200 μmol/L throughout the follow‐up period. An established definition of classic PKU is a maximum untreated blood phenylalanine level above 1200 μmol/L. [22] Therefore, for children with a chart‐based diagnosis of classic PKU but without any chart‐documented values of blood phenylalanine above 1200 μmol/L, we explored other potential explanations for the chart‐based diagnosis: (i) presence of a sibling with a chart‐based diagnosis of classic PKU; and (ii) becoming a patient at a participating metabolic centre after the diagnostic period (i.e., missing data from early infancy). Both characteristics were recorded in the study dataset. We excluded children with no diagnostic classification documented in their medical chart from analyses stratified by diagnostic classification.

### 2.4 Statistical Analysis

We used descriptive statistics (counts, proportions, means/standard deviations (SD) or medians/interquartile ranges (IQR), as appropriate) to summarize demographic data, newborn screening status, and receipt of pharmacotherapy. To protect the confidentiality of patients, we did not report results where fewer than 5 children contributed data. Missing data were minimal (≤ 6%). For any analysis containing missing data, we used casewise deletion. All data analysis procedures were performed using SAS software version 9.4 (SAS Institute Inc., Cary, NC), and Microsoft Excel.

#### 2.4.1 Metabolic Control During the First Month of Life for Children With Classic PKU

We calculated the time needed to achieve initial metabolic control during the first month of life for children with classic PKU as the child's age (in days) at which the first measured value of blood phenylalanine reached the therapeutic range of 120–360 μmol/L. We also calculated the percentage of children with 1 or more, 2 or more, or 3 or more blood phenylalanine values below the lower end of the therapeutic range, < 120 μmol/L.

Due to anticipated heterogeneity in pre‐treatment blood phenylalanine levels and the timing of instituting treatment, and consequential challenges in interpreting related results, we excluded data from the first month of life from children with less severe PAH deficiency from these analyses.

#### 2.4.2 Metabolic Control Beyond the First Month of Life for the Full Cohort

First, to examine the distribution of a child's blood phenylalanine levels in relation to the therapeutic range (i.e., 120–360 μmol/L) after the first month of life, we calculated the mean blood phenylalanine level by diagnostic classification (classic PKU or less severe PAH deficiency) and clinically meaningful age group (> 1–6 months, > 6–12 months, > 1–2 years, > 2–3 years, > 3–4 years, > 4–5 years, > 5–6 years, > 6–7 years, > 7 years). To achieve this, we fit a mixed effects regression model that accounted for the autocorrelation of repeated measurements within children followed longitudinally and included diagnostic classification and age group, as well as their interaction, as independent variables. We reported the least square means of blood phenylalanine levels alongside their 95% confidence intervals (CIs) and visually presented them relative to the therapeutic range. To evaluate the potential impact of misclassification of diagnostic category, we conducted a post hoc sensitivity analysis of our mixed effects model results, excluding all blood phenylalanine values for children diagnosed with classic PKU whose data did not meet the established definition of classic PKU and for whom we did not have an alternative explanation for the diagnostic classification.

Second, we calculated the proportion of children considered to be in metabolic control during the follow‐up period, by diagnostic classification and age group [5, 6]. We adapted Hartnett and colleagues [23], definition of metabolic control: having more than 60% of blood phenylalanine values within the therapeutic range (i.e., 120–360 μmol/L). Since this definition does not distinguish between values above or below the therapeutic range, we conducted a sensitivity analysis to investigate blood phenylalanine values above the therapeutic range (i.e., > 360 μmol/L) as an indicator of being outside of metabolic control. We reported the proportion of children with more than 60% of their blood phenylalanine values below or equal to 360 μmol/L (i.e., within or below the therapeutic range), for the whole sample and categorized by diagnostic classification and age group. Furthermore, we calculated the proportion of blood phenylalanine values that were outside the therapeutic range (i.e., either < 120 and > 360 μmol/L) for each child, the proportion of values that were above 360 μmol/L, and the proportion below 120 μmol/L.

#### 2.4.3 Frequency of Clinical Monitoring of Metabolic Control for the Full Cohort

We calculated the rate of blood phenylalanine testing and the rate of communication (in‐person and virtual clinical interactions with providers) per child‐month. To calculate these rates, we divided the total count of events (blood phenylalanine tests or clinical interactions) by the total number of follow‐up months for clinically relevant age groups (i.e., 0–1 months, > 1–12 months, > 1–7 years, and > 7 years) [5].

## 3 Results

### 3.1 Participant Characteristics

From 215 cohort participants (49% females) with a diagnosis of PAH deficiency, 92 were classified as having classic PKU in the medical chart, 25 with mild PKU, and 85 with mild hyperphenylalaninemia (Table 1); the latter two categories were grouped as “less severe PAH deficiency” for some analyses. Thirteen children (6%) did not have a diagnostic classification and were excluded from analyses that were stratified by diagnostic classification. During the follow‐up period, 32% of children with classic PKU received sapropterin and 19% continued treatment during follow‐up, 48% of children with mild or moderate PKU received sapropterin and 36% continued treatment during follow‐up, and 7% of children with mild hyperphenylalaninemia received and continued sapropterin during follow‐up. The median age of the child at the start of treatment was 32 and 30 months, for those with classic PKU or less severe PAH deficiency, respectively. No patients received other medications (e.g., pegvaliase) to manage their PKU symptoms.

**TABLE 1:** Characteristics of included participants.

Characteristic	*n*	%
Sex assigned at birth (*n* = 215)		
Male	109	51
Female	106	49
Year of birth (*n* = 215)		
2006–2007	43	20
2008–2009	34	16
2010–2011	36	17
2012–2013	48	22
2014–2015	54	25
Treatment centre at time of consent (*n* = 215)		
Alberta Children's Hospital (Calgary)	11	5
BC Children's Hospital (Vancouver)	36	17
Children's Hospital of Eastern Ontario (Ottawa)	14	7
Children's Hospital – Health Science Centre Winnipeg	11	5
Children's Hospital – London Health Sciences Centre	16	7
Stollery Children's Hospital (Edmonton)	12	6
Hospital for Sick Children (Toronto)	66	31
Izaak Walton Killam Health Centre (Halifax)	9	4
L'Hôpital de Montréal pour enfants du centre universitaire de santé McGill	11	5
McMaster Children's Hospital—Hamilton Health Sciences	20	9
Other centres^a^	9	4
Chart‐reported PAH deficiency diagnostic category (*n* = 215)		
Classic PKU	92	43
Less severe PAH deficiency	110	51
Mild or moderate PKU	25	12
Mild Hyperphenylalaninemia (HPA)	85	40
Not recorded in chart	13	6
Identified by newborn screening (*n* = 215)		
Yes	207	96
No or unknown^b^	8	4
Received Sapropterin for treatment of PAH deficiency		
Entire cohort (*n* = 215)	47	22
Classic PKU (*n* = 92)	29	32
○ Remained on sapropterin during follow‐up	17	19
○ Discontinued sapropterin	12	13
Less severe PAH deficiency (*n* = 110)	18	16
Mild or moderate PKU (*n* = 25)	12	48
○ Remained on sapropterin during follow‐up	9	36
○ Discontinued sapropterin	3	12
Mild Hyperphenylalaninemia (HPA) (*n* = 85)	6	7
○ Remained on sapropterin during follow‐up	6	7
○ Discontinued sapropterin	0	0
Age of child when Sapropterin was started (*n* = 47)	Median (months)	IQR
Classic PKU	32	38
Less Severe PAH deficiency	30	37

Abbreviation: IQR, interquartile range.

^a^ Other centres included: Kingston General Hospital and Le Centre hospitalier universitaire de Sherbrooke.

^b^ Cases not identified by newborn screening because child: had moved from a different country without a newborn screening program (*n* < 5), had been primarily identified because of an older sibling (i.e., had a diagnostic test before newborn screening or identified prenatally) (*n* < 5), diagnosis was ascertained at a non‐participating center (*n* < 5), or information was not available about their newborn screening status (*n* < 5).

### 3.2 Diagnostic Classification of Classic PKU

Among the 92 children with a chart diagnosis of classic PKU, 64 (69.5%) had at least one recorded blood phenylalanine value above 1200 μmol/L at any age during the study follow‐up period (not shown). Of the remaining 28 children, 10 were reported to have one or more siblings with a chart diagnosis of classic PKU, and 3 had missing diagnostic and monitoring data during early infancy, for example, due to joining a participating metabolic centre after this period. For the remaining 15 children, we were unable to identify the criteria used to diagnose classic PKU.

### 3.3 Metabolic Control During the First Month of Life

Among 70 children in the cohort diagnosed with classic PKU whose medical chart included at least three blood phenylketonuria values during the first month of life, a first recorded blood phenylalanine level within the therapeutic range (i.e., 120–360 μmol/L) was observed at a median age of 15 days of life (IQR 11) (Table 2). Nearly three‐quarters of children with classic PKU in this analysis (74.3%) had at least one blood phenylalanine value below the target therapeutic range (i.e., < 120 μmol/L), 57.1% had 2 or more values below the target range, and 32.9% had 3 or more values below 120 μmol/L.

**TABLE 2:** Metabolic control during the first month of life for children with classic PKU.

	*n* ^a^	Median (days)	Range (days)	IQR (days; 25th—75th percentile)
Age at first blood phenylalanine value within the therapeutic range (i.e., 120–360 μmol/L)	70	15	3–107	11 (12–23)

	*N* ^a^	n	%	95% CI
Proportion of children with blood phenylalanine values below the therapeutic range (i.e., < 120 μmol/L)				
≥ 1 values	70	52	74.3	64.0–84.5
≥ 2 values	70	40	57.1	45.5–68.7
≥ 3 values	70	23	32.9	21.9–43.9

Abbreviations: CI, confidence interval; IQR, interquartile range.

^a^ Total number of children with classic PKU who had 3 or more blood phenylalanine measurements recorded in the medical chart from a participating clinic within the first month of life.

### 3.4 Metabolic Control After the First Month of Life

Our longitudinal mixed effects model included 79 children with classic PKU and 85 with less severe PAH deficiency who had more than one blood phenylalanine value beyond the first month of life. We found a statistically significant relationship between blood phenylalanine level and both diagnostic classification and age group (*p* < 0.001). The interaction between these two variables was not statistically significant (*p* = 0.26), but was retained in our model to calculate the least squares mean blood phenylalanine levels for each of the disease classifications within age groups.

**FIGURE 1:**
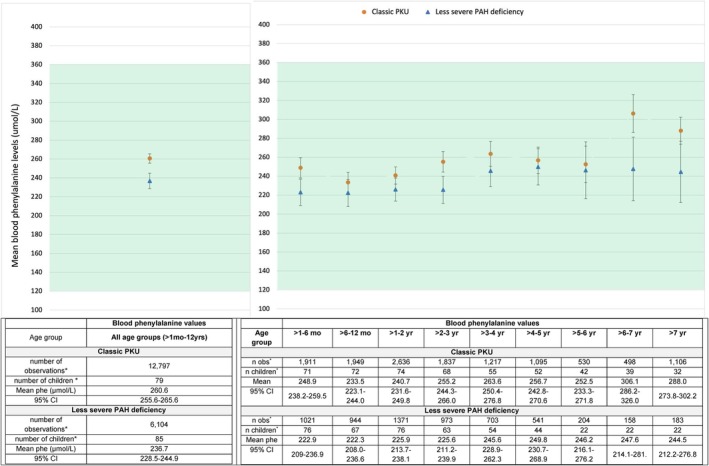
(a: Left) Mean blood phenylalanine levels (μmol/L) averaged across all age categories, by diagnostic category. (b: Right) Age‐specific blood phenylalanine levels (μmol/L), by diagnostic category. Mo, month; phe, phenylalanine; yr, year. * number of observations (*n* obs) = total number of blood phenylalanine test values (multiple values within the same child), number of children (*n* children) = number of unique children contributing data per age group (the same child could have contributed data to multiple age groups). Least square mean blood phenylalanine (μmol/L) with 95% confidence intervals shown, excluding values from the first month of life to account for pre‐post treatment institution variability; “Less severe PAH deficiency” includes mild and moderate PKU and mild hyperalaninemia (HPA).

From this model, the estimated cohort mean blood phenylalanine level across all age categories was within the target therapeutic range (i.e., 120–360 μmol/L) for those with classic PKU (mean 260.6 μmol/L; 95% CI 255.6–265.6) and those with less severe PAH deficiency (mean 236.7 μmol/L; 95% CI 228.5–244.9) (Figure 1a). This held true for children in each age group (Figure 1b). Children with classic PKU had higher mean phenylalanine levels relative to those with less severe PAH deficiency across age groups (difference in least square means = 23.9 μmol/L; 95% CI 14.3–33.4; *p* < 0.0001). In both diagnostic categories, there was evidence of an increase in mean blood phenylalanine with age after 12 months of life and with some fluctuations (Figure 1b and Appendix 1). This increase was most prominent in the older age groups: mean blood phenylalanine levels regardless of diagnostic classification were the highest among those aged 6 to 7 years (mean = 276.9 μmol/L; 95% CI 257.4–269.4) and older than 7 years (mean = 266.3 μmol/L; 95% CI 248.6–283.9) (Appendix 1).

The mean blood phenylalanine level of the 13 children who had no disease classification in their medical charts was also in the target therapeutic range (257.4 μmol/L; 95% CI 235.8–278.9).

Our post hoc sensitivity analysis showed that excluding blood phenylalanine values (1775/12797) from 15 children diagnosed with classic PKU for whom there were no blood phenylalanine levels above 1200 μmol/L, and for whom we were unable to find an alternative explanation for their diagnostic classification, had no substantial impact on our findings with respect to metabolic control (Appendix 1).

**FIGURE 2:**
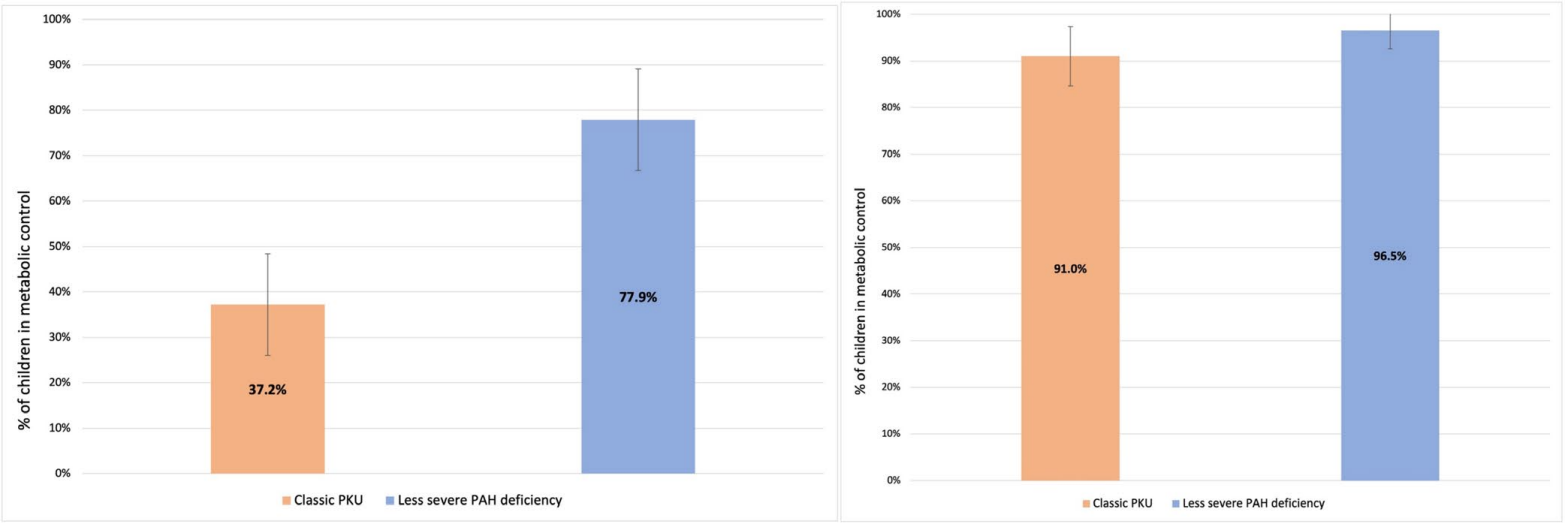
(a: Left). Metabolic control: Children with greater than 60% of blood phenylalanine values within the therapeutic range, throughout follow‐up, for children with classic PKU (*n* = 79) and less severe PAH deficiency (*n* = 85). A child is considered in metabolic control if more than 60% of their blood phenylalanine values beyond the first month of life are within the therapeutic range (i.e., 120–360 μmol/L) [23]; Children who contributed 3 or more blood phenylalanine values were included in the analysis. (b: Right). Sensitivity analysis: Children with greater than 60% of blood phenylalanine values within or below the therapeutic range, throughout follow‐up, for children with classic PKU (*n* = 79) and less severe PAH deficiency (*n* = 85). Examining only the upper limit of the therapeutic range (i.e., ≤ 360 μmol/L) to define metabolic control. A child is considered in metabolic control if more than 60% of their blood phenylalanine values beyond the first month of life are ≤ 360 μmol/L; Children who contributed 3 or more blood phenylalanine values were included in the analysis.

Considering all blood phenylalanine values after the first month of life and through the end of follow‐up, 29 of 79 children with classic PKU (37.2%) were considered to be in metabolic control compared to 67 of 85 children with less severe PAH deficiency (77.9%) (figure 2a, difference between groups χ^2^ = 27.9; *p* < 0.0001). In our sensitivity analysis examining the percentage of children with more than 60% of their blood phenylalanine levels within *or below* the therapeutic range (i.e., ≤ 360 μmol/L), more than 90% of children with classic PKU, and of those with less severe PAH deficiency, were in metabolic control by this revised definition; the difference between the two groups was not statistically significant (*p* = 0.14) (Figure 2b).

**FIGURE 3:**
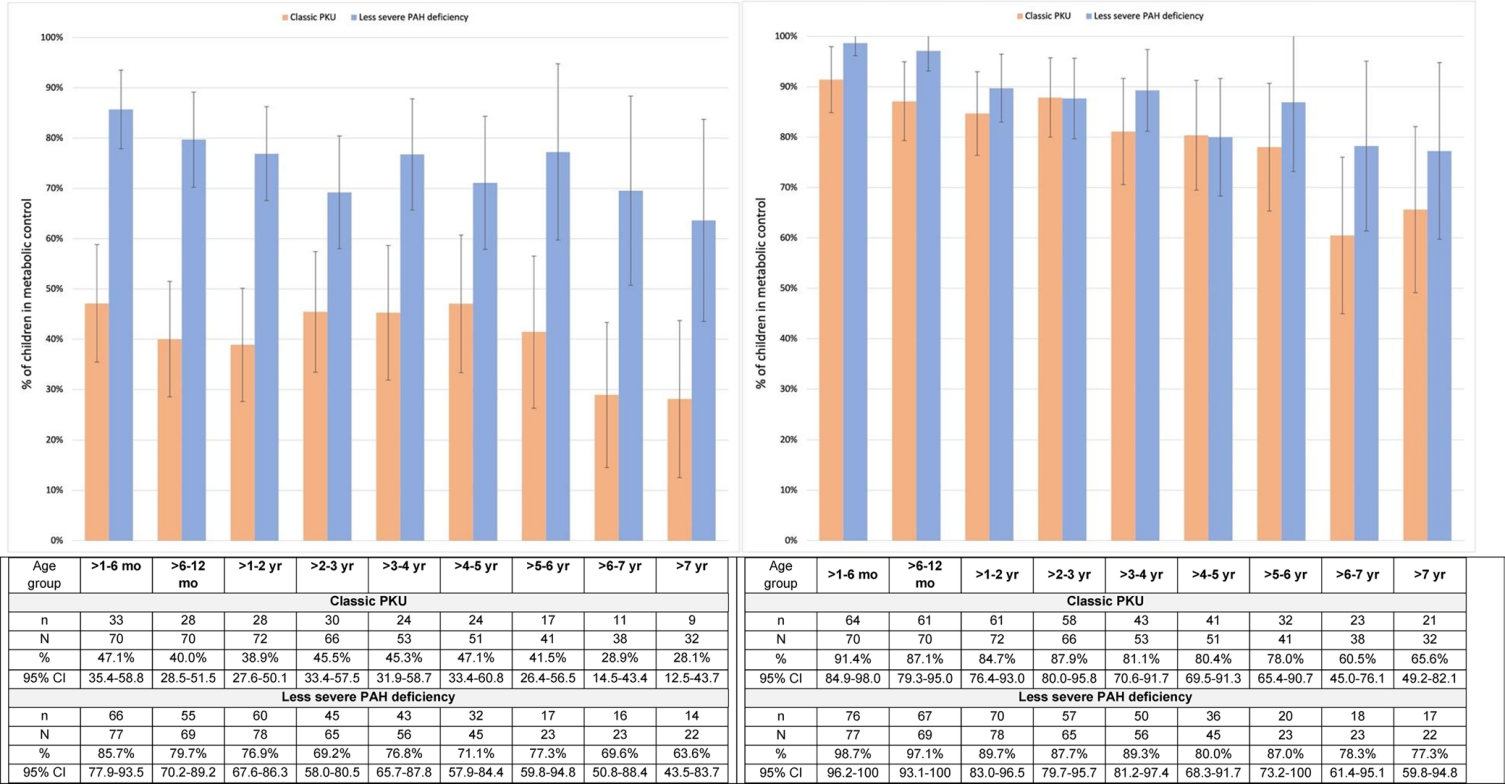
(a: Left). Metabolic control: Children with > 60% of blood phenylalanine values within the therapeutic range, for each age group, for children with classic PKU and less severe PAH deficiency. A child is considered in metabolic control if more than 60% of their blood phenylalanine values beyond the first month of life are within the therapeutic range (i.e., 120–360 μmol/L) (Adapted from Hartnett et al. [23]); Children who contributed 3 or more blood phenylalanine values and were included in the analysis; *n* = number of children in metabolic control; *N*=number of children in the age group. (b: Right). Sensitivity analysis: Children with > 60% of blood phenylalanine values within or below the therapeutic range, for each age group, for children with classic PKU and less severe PAH deficiency. Examining only the upper limit of the therapeutic range (i.e., ≤ 360 μmol/L) to define metabolic control; A child is considered in metabolic control if more than 60% of their blood phenylalanine values beyond the first month of life are ≤ 360 μmol/L; Children who contributed 3 or more blood phenylalanine values and were included in the analysis; *n* = number of children in metabolic control; *N*=number of children in the age group.

When metabolic control was examined across age groups, the percentage of children with classic PKU who were in metabolic control was lower in every age group compared to those with less severe PAH deficiency (Figure 3a). Among children with classic PKU, the lowest proportions meeting the metabolic control criteria were in the oldest age categories, that is, among children aged 6–7 years (28.9%; 95% CI 14.5, 43.4) and older than 7 years (28.1%; 95% CI 12.5, 43.7). The lowest proportions of children with less severe PAH deficiency meeting the metabolic control criteria were between 2 and 3 years of age (69.2%; 95% CI 58.0–80.5), 6–7 years (69.6%; 95% CI 50.8–88.4), and older than 7 years (63.6%; 95% CI 43.5–83.7). Our sensitivity analysis showed that the majority of children in both diagnostic classification groups and across all age categories had more than 60% of their blood phenylalanine levels within or *below* the therapeutic range (i.e., ≤ 360 μmol/L) (Figure 3b). A lower proportion of older children with classic PKU in the cohort (> 6–7 years and > 7 years) met this criterion (60.5% and 65.6%, respectively) relative to other age groups and relative to those with less severe PAH deficiency, although confidence intervals were wide.

The percentage of blood phenylalanine values outside of the target therapeutic range after the first month of life during the follow‐up period was higher among children with classic PKU (median per child = 43.6%; range 9.1–88.9; IQR = 18) compared to those with less severe PAH deficiency (median per child = 15.3%; range 0–100; IQR = 33.2) (Appendix 2; Table A2.1). Children with classic PKU also had higher proportions of values above 360 μmol/L (23.7% and 4.9%) and below 120 μmol/L (21.7% and 2.6%) compared to children with less severe PAH deficiency (Appendix 2; Table A2.1). Similarly, when stratifying results by metabolic control status, we found that children with classic PKU who met the definition of metabolic control had a higher percentage of values outside the therapeutic range as well as values above 360 μmol/L and below 120 μmol/L compared to those with less severe PAH deficiency (Appendix 2; Tables A2.2 and A2.3).

### 3.5 Frequency of Clinical Monitoring

The rate of blood phenylalanine testing for children with classic PKU declined stepwise with age from approximately 9 tests per month during the first month of life to approximately 1 test per month in children older than 7 years of age (Table 3). Similarly, testing rates among children with less severe PAH deficiency declined stepwise with age from a rate of approximately 3 tests per child per month during the first month of life to an average of one test every 3 months among children older than 7 years of age (Table 3).

**TABLE 3:** Frequency of clinical monitoring of metabolic control by age and diagnostic category.

	Rate of blood phenylalanine testing (per child‐month)^a^			
	Age group			
	0–1 month	> 1–12 months	> 1–7 years	> 7 years
Diagnostic category				
Classic PKU	8.98 (*n* = 82)	4.40 (*n* = 82)	1.90 (*n* = 82)	1.07 (*n* = 33)
Less severe PAH deficiency	3.28 (*n* = 103)	1.47 (*n* = 103)	0.77 (*n* = 99)	0.32 (*n* = 26)

	Rate of communication with metabolic centres (per child‐month)^b^			
	Age group			
	0–1 month	> 1–12 months	> 1–7 years	> 7 years
Diagnostic category				
Classic PKU	1.59 (*n* = 82)	0.54 (*n* = 82)	0.17 (*n* = 82)	0.15 (*n* = 33)
Less severe PAH deficiency	0.88 (*n* = 103)	0.26 (*n* = 103)	0.11 (*n* = 99)	0.09 (*n* = 26)

^a^ Calculated using the formula: total count of tests divided by the sum of individually contributed months of follow‐up (interpreted as average number of tests per child per month).

^b^ Calculated using the formula: total count of in‐person or virtual interactions (including phone calls) divided by the sum of individually contributed months of follow‐up (interpreted as average interactions per child per month).

Rates of communication with metabolic centres among children with classic PKU, similar to blood testing trends, declined with age from a rate of approximately 1.5 interactions per child per month during the first month of life to 0.15 interactions per child per month among children older than 7 years (Table 3). Among children with less severe PAH deficiency, communication rates declined from a rate of 0.88 interactions per child per month during the first month of life to 0.09 interactions per child per month among children older than 7 years (Table 3).

## 4 Discussion

This study analyzed longitudinal data on blood phenylalanine levels as the primary metabolic control outcome for 215 children with PAH deficiency (classified based on the assumed severity of their symptoms as classic PKU or less severe PAH deficiency) born between 2006 and 2015 and treated at one of 12 Canadian pediatric metabolic centres. Several cohort studies have followed children with PAH deficiency longitudinally for clinical outcomes such as metabolic control [24, 25, 26, 27], growth [28], and neuropsychological development [25, 29]. To our knowledge, our study is the first birth cohort study to analyze metabolic control outcomes during the first month of life and throughout childhood in Canada.

Our findings show that during the first month of life, children with classic PKU took a median of 15 days for their blood phenylalanine values to reach the target therapeutic range (i.e., 120–360 μmol/L). However, values below this therapeutic range (i.e., < 120 μmol/L) occurred in up to 74.3% of children. One reason for the high frequency of values below the therapeutic range may be a reaction to a phenylalanine wash out prescribed in cases with very high pre‐treatment blood phenylalanine levels in children with classic PKU. Another possible contributor to fluctuations in, and low values of, blood phenylalanine levels could be changes in dietary phenylalanine intake during the first month of life, in particular, over restriction of phenylalanine intake; however, causal evidence remains limited. A cohort study published in 1991 of early‐treated patients with PKU found significant variability in mean blood phenylalanine levels between children in different pediatric practices early in childhood, which might suggest heterogeneity in how metabolic control was defined and maintained by different clinicians [26]. Future research should explore clinician perspectives on and real‐world implementation of current PKU management guidelines, particularly regarding the lower end of the therapeutic range. While treatment to achieve metabolic control at an early age is essential to prevent negative long‐term neurologic sequelae, avoiding the over restriction of phenylalanine intake and its potential contribution to low blood phenylalanine values is crucial due to its potential role for suboptimal growth outcomes [30].

Our findings suggest similar variability in blood phenylalanine levels beyond the first month of life and throughout the follow‐up period: while mean blood phenylalanine levels were within the recommended therapeutic range (i.e., 120–360 μmol/L) across all age groups and classifications, we found that fewer than 40% of children with classic PKU in our cohort met the criterion for being in metabolic control over the follow‐up period [5, 23], whereas more than 75% of those with less severe PAH deficiency did. Compared to children with less severe PAH deficiency, the proportion of blood phenylalanine values among children with classic PKU that were above 360 μmol/L was greater, as was the proportion of values below 120 μmol/L. Previous studies suggest that homeostasis of blood phenylalanine levels may be more closely correlated with long‐term outcomes than average values over time [31, 32]. In particular, intelligence quotient and executive functioning, which have been identified as important outcomes by family and clinical caregivers of children with PKU [33], have been shown to be influenced by blood phenylalanine variability [31, 32]. This highlights the importance of investigating blood phenylalanine variability in this population rather than focusing solely on measures of central tendency as an indicator of metabolic control. New innovations, such as home blood phenylalanine monitors [34], may offer children with PAH deficiency the opportunity to reach metabolic control faster and reduce variability in blood phenylalanine.

Our study showed a trend of increased blood phenylalanine levels starting as early as 6 years of age. This finding is corroborated by other studies; a large retrospective cohort study of 1323 PAH deficiency patients across nine European countries (aged 1 to 57 years) showed an increase in blood phenylalanine levels and variability with age across all diagnostic classifications [27]. Similarly, a cross‐sectional study of 30 pediatric patients with PKU in the United States found a statistically significant correlation between increasing age of children 5 to 16 years old and blood phenylalanine levels (correlation coefficient *r* = 0.44; *p* = 0.02) [35]. Another cross‐sectional study of 105 PKU children treated in Spain found a statistically significant increase in blood phenylalanine levels with increased age (categorized, < 6 years, 6–12 years, and > 12 years) (*p* < 0.001) [36]. A retrospective cohort study of 37 patients with PKU in the United States who were on sapropterin found an increase in blood phenylalanine levels of 14.5 μmol/L per year from ages 1.5 to 32 years (*p* < 0.0001) [37].

Increased phenylalanine levels with age may be related to challenges in maintaining metabolic control in the face of changing growth rates [35] and/or periods of higher protein catabolism due to illnesses and infections, which are more prominent as children enter school age [38, 39]. The PKU medical diet is known to be arduous; treatment adherence and frequent clinic visits may be difficult for children and their caregivers [40, 41]. Several studies have observed a decrease in adherence to the PKU medical diet with increasing child age [18, 23, 42, 43], which may relate to changes in the social environment (e.g., school entry), increased child independence in eating habits, and/or a decreased frequency of treatment monitoring. A longitudinal retrospective study of 75 children with PKU suggested that higher blood phenylalanine levels among children 6 to 8 years old may predict discontinuation of PKU treatment in adolescence [44]. The influence of child, caregiver, or other factors is unclear, although child attributional style (i.e., internal vs. external locus of control) and parenting strategies and attitudes may be associated with metabolic control [45]. Further research is needed to understand whether children with higher blood phenylalanine levels at an early age are at risk of long‐term challenges with dietary adherence, and if so, to identify interventions that may help to mitigate this risk and support families as their children transition into adolescence and the potential cost implications of such interventions.

Among children with classic PKU and those with less severe PAH deficiency, we observed rates of monitoring decline with increasing age. More frequent blood phenylalanine measurements are recommended for children with higher blood phenylalanine levels and more severe symptoms [5, 6]. In our study, the frequency of clinical monitoring of children with classic PKU aligned with the Genetic Metabolic Dietitians International GMDI [5, 6] guidelines. Among children with less severe PAH deficiency, monitoring was less frequent compare to children with classic PKU. Children with less severe PAH deficiency, such as mild PKU and mild hyperphenylalaninemia, may reach and maintain metabolic control with a mild reduction in phenylalanine intake and, therefore, may not require frequent monitoring.

We recommend, however, that future research explore the benefits of frequent monitoring for children not in metabolic control. In our study, we found that more than 60% of children with classic PKU aged > 1 month and 22% of children with less severe PAH deficiency did not meet the criterion for being in metabolic control. The negative health consequences of high blood phenylalanine levels among children with less severe PAH deficiency cannot be ignored. More research is needed to assess whether children with higher variability in blood phenylalanine levels may benefit from increased frequency of monitoring, which has been found to predict the success of PAH deficiency treatment [5, 18]. Furthermore, our data collection period occurred before the COVID‐19 pandemic, and therefore was not subject to any related interruptions to health care or monitoring. Future research ought to consider the consequential healthcare provision changes brought by this pandemic and how certain alternative models of care, such as telemedicine and integrated care approaches, have affected treatment and monitoring for children with PAH deficiency, in Canada and globally.

To our knowledge, our study is the first Canadian cohort study to follow children with PAH deficiency longitudinally from birth and up to 12 years of age. As most children with PAH deficiency in Canada receive care at regional metabolic centres, the study sample likely reflects a representative population of Canadian pediatric patients with PAH deficiency. We used robust statistical analyses to explore the mean blood phenylalanine levels among children across age groups and diagnostic classifications while accounting for the autocorrelation of repeated measurements within children and also explored metabolic control over time. This study has limitations. Our data was retrospective and chart‐abstracted; we relied on children's chart‐reported diagnosis of PAH deficiency, but diagnostic criteria may have differed between treatment centres. For example, 15 children with classic PKU did not have blood phenylalanine levels above 1200 μmol/L, the established definition for this diagnostic classification [22], and we could not otherwise verify the classification. We postulate that clinical advancements, such as early reporting of newborn screening, faster diagnosis and treatment initiation, and more frequent monitoring may have helped prevent children with classic PKU from reaching high phenylalanine levels during the time of diagnosis. Nonetheless, we found through a post hoc sensitivity analysis that mean blood phenylalanine levels and the difference in mean levels between children with classic PKU and less severe PAH deficiency did not change when excluding values from these children. Furthermore, the method of blood sample collection (i.e., dried blood spot and plasma draw) was not consistently recorded across all participants, so we treated blood phenylalanine values as a single group for the purposes of analysis. We could not distinguish between children with mild or moderate PKU and mild hyperphenylalaninemia due to unclear disease classification criteria across centers and the unavailability of diagnostic test values for a significant proportion of participants. Therefore, we grouped these children under a single classification for analysis (i.e., “less severe PAH deficiency”). We recognize that this group may exhibit heterogeneity in their blood phenylalanine variability and the timing of treatment initiation and monitoring. Ideally, additional information such as phenylalanine tolerance and correlation with genotype would be used in classification of PAH deficiency. It would be important to distinguish between mild PKU and mild phenylalaninemia in future research on clinical outcomes such as growth or neurocognitive development.

Lastly, we anticipate that the frequency of monitoring may have increased around times of marked high blood phenylalanine values, which may be caused by catabolic illness (e.g., the flu season) or changes in adherence to the PKU medical diet. Our dataset lacked information on concurrent morbidities and diet adherence, which limited our ability to investigate these potential factors. While our mixed model accounted for the autocorrelation of children's blood phenylalanine measures, controlling for repeatedly higher or lower values within a close timeframe, future research should explore the temporal patterns of metabolic control and frequency of monitoring in relation to these variables in order to better understand the circumstances for, and make recommendations on any changes to the frequency of, clinical monitoring of all children with PAH deficiency.

## 5 Conclusion

In this retrospective birth cohort study including 215 children (aged 0–12 years) with PAH deficiency in Canada, we found that average phenylalanine values were generally within the recommended therapeutic range (i.e., 120–360 μmol/L) across age groups and diagnostic classification categories. Children with classic PKU, however, frequently experienced blood phenylalanine levels above and below the therapeutic range at all ages; their mean levels were higher compared to children with less severe PAH deficiency, and values tended to increase with increasing age, starting as early as 6 years of age. While the frequency of clinical monitoring of children with classic PKU, via blood phenylalanine testing and communication with metabolic centres, was aligned with recommendations, this frequency declined with age for all children with PAH deficiency. Our findings demonstrate the degree of blood phenylalanine variability among children with PAH deficiency. They highlight a need for particular attention to fluctuations in metabolic control for children diagnosed with classic PKU and older children, regardless of diagnostic classification, as well as a need for the identification of supports for children with PAH deficiency and their caregivers towards minimizing fluctuations in metabolic control and associated prevention of suboptimal outcomes.

## Author Contributions

N.Y., B.K.P., S.S.‐I., J.J.M., M.L., J.B.K., K.W., A.F., M.I.‐F., J.L., S.M.‐A., A.P., C.P., A.Sc., Y.T., GH, H.V., V.A., A.V., C.R.‐G., A.A.M., and P.C. conceptualized, designed, and planned the study. N.Y., B.K.P., S.S.‐I., J.J.M., K.T., M.L., J.B.K., A.F., S.G., M.I.‐F., S.M.‐A., A.P., C.P., A.Sc., Y.T., G.H., V.A., A.V., A.C.Y., C.R.‐G., A.A.M., and P.C. acquired the data. N.Y., A.Sa., B.K.P., S.H., K.T., M.P., and P.C. analyzed the data. N.Y., A.S., B.K.P., S.S.‐I., J.J.M., S.H., K.T., M.P., M.P., A.J.C., J.B.K., K.W., A.F., S.G., M.I.‐F., J.L., S.M.‐A., A.P., C.P., A.S., Y.T., H.V., V.A., A.V., A.C.Y., C.R.‐G., A.A.M., and P.C. interpreted the data. N.Y., A.S., B.K.P., S.S.‐I., K.T., A.J.C., and P.C. drafted the manuscript. All authors critically revised the manuscript and approved the final manuscript.

## Ethics Statement

The cohort study was approved by the research ethics boards of all participating sites, including: Children's Hospital of Eastern Ontario Research Ethics Board, Conjoint Health Research Ethics Board at the University of Calgary, Hamilton Integrated Research Ethics Board, IWK Research Ethics Board, McGill University Health Centre Research Ethics Committee (for Montreal Children's Hospital and Le centre hospitalier universitaire Sherbrooke), Newfoundland and Labrador Health Research Ethics Board, Ottawa Health Science Network Research Ethics Board, Queen's University Health Sciences and Affiliated Teaching Hospitals Research Ethics Board, The Hospital for Sick Children's Research Ethics Board, University of Alberta Health Research Ethics Board—Health Panel, University of British Columbia Children's and Women's Research Ethics Board, University of Manitoba Health Research Board, and Western University Health Science Research Ethics Board.

## Consent

We obtained written informed consent from caregivers of patients included in the study.

## Conflicts of Interest

A.P. has participated in Dietitian advisory boards for: Ultragenyx (June 2024, April 2022 and March 2021) and Horizon (April 2022, April 2021). J.J.M. has received funding for nursing support from Biomarin for follow‐up of PKU and Morquio A patients, and he has received consulting fees from PTC Therapeutics. M.I.‐F. was a site co‐investigator for clinical trials with PTC Therapeutics. P.C. has received grant support from Biomarin, Cambrooke, Vitaflo, Nutricia, and National Food Distribution Centre. The remaining authors have no competing interests to declare.

## Supporting information


**Data S1:** Appendix 1 Supporting information.

**Data S2:** Appendix 2 Supporting information.

## Data Availability

We are not able to share record‐level data based on the approved research ethics board approval and associated privacy laws. The code used to analyze the findings is available from the corresponding author upon reasonable request.
