# Supplementary material for: Metabolic Control and Frequency of Clinical Monitoring Among Canadian Children With Phenylalanine Hydroxylase Deficiency: A Retrospective Cohort Study
Source: JIMD Rep. 2025 Sep 1;66(5):e70042. doi: 10.1002/jmd2.70042 (PMC12401562; doi:10.1002/jmd2.70042)
Supplement: Supplementary file 1 — Data S1: Appendix 1 Supporting information. [file JMD2-66-e70042-s001.pdf]

Appendix 1. Additional descriptive analyses by age group

Table A1.1 Mean blood phenylalanine level (µmol/L), by age group

Age group	Estimate[a]	Standard error	95% CI
>1-6 mo	235.9	4.47	227.1 - 244.7
>6-12 mo	227.9	4.52	219.1 - 236.8
>1-2 yr	233.3	3.87	225.7 - 240.9
>2-3 yr	240.4	4.59	231.4 - 249.4
>3-4 yr	254.6	5.42	243.9 - 265.2
>4-5 yr	253.3	6.02	241.5 - 265.1
>5-6 yr	249.4	9.09	231.5 - 267.2
>6-7 yr	276.9	9.93	257.4 - 269.4
>7 yr	266.3	8.99	248.6 - 283.9

[a] Least square means from linear mixed effect model accounting for the autocorrelation of repeated measurements within children followed longitudinally and categorized by age group only

Table A1.2 Sensitivity analysis: Excluding blood phenylalanine values (1775 of 12,797 observations) from 15 children diagnosed with classic PKU for whom there were no blood phenylalanine levels above 1200 µmol/L and we were unable to find an alternative explanation for their diagnostic classification

Age group	**>1-6 mo**	**>6-12 mo**	**>1-2 yr**	**>2-3 yr**	**>3-4 yr**	**>4-5 yr**	**>5-6 yr**	**>6-7 yr**	**>7 yr**
Diagnostic category	**Classic PKU**								
No. observations	1581	1612	2271	1602	1084	942	453	420	1042
Mean	254.7	234.8	243.1	256.9	264.2	263.3	252.6	317.8	288.5
95% CI	242.6-266.8	222.9-246.7	232.9-253.2	244.9-268.9	249.7-278.7	247.8-278.7	231.2-274.1	295.5-340.2	273.3-303.6

	**Difference in mean blood phenylalanine values when excluding N=1775 observations from 15 children with unclear diagnosis criteria**								
Age group	**>1-6 mo**	**>6-12 mo**	**>1-2 yr**	**>2-3 yr**	**>3-4 yr**	**>4-5 yr**	**>5-6 yr**	**>6-7 yr**	**>7 yr**
Diagnostic category	**Classic PKU**								
Observation difference	330	337	365	235	133	153	77	78	64
Mean difference	-5.83	-1.31	-2.37	-1.79	-0.66	-6.53	-0.1	-11.72	-0.47
	Average difference= -3.42 (SD 3.87); 95% CI -6.83, -0.01								

Table depicts number of excluded observations and the difference in the mean blood phenylalanine levels between those included and excluded from the mixed model
