# Supplementary material for: Metabolic Control and Frequency of Clinical Monitoring Among Canadian Children With Phenylalanine Hydroxylase Deficiency: A Retrospective Cohort Study
Source: JIMD Rep. 2025 Sep 1;66(5):e70042. doi: 10.1002/jmd2.70042 (PMC12401562; doi:10.1002/jmd2.70042)
Supplement: Supplementary file 2 — Data S2: Appendix 2 Supporting information. [file JMD2-66-e70042-s002.pdf]

Appendix 2. Sensitivity analysis of blood phenylalanine values outside the therapeutic range, whole sample and stratified by in or not in metabolic control

Table A2.1 Blood phenylalanine values outside the therapeutic range of 120-360 μmol/L, above 360 μmol/L, and below 120 μmol/L among all blood phenylalanine values, per child for all children

Per child, from one month of age to end of follow-up	All children					
	Classic PKU (n=80)			Less severe PAH deficiency (n=84)		
	Median # (range)	Median % (range)	IQR (25th-75th percentile)	Median # (range)	Median % (range)	IQR (25th-75th percentile)
Values **outside 120-360 μmol/L**	56.5 (2-242)	**43.6** (9.1-88.9)	18 (35.2-53.2)	6.0 (0-133)	**15.3** (0.0-100)	33.2 (3.3-36.5)
Values **above 360 μmol/L** [a]	22.5 (1-135)	**23.7** (1.0-88.9)	17.4 (11.2-28.6)	3.0 (0-87)	**4.9** (0.0-60.9)	17.4 (0.0-17.4)
Values **below 120 μmol/L** [b]	33.5 (0-107)	**21.4** (0.0-46.5)	13.8 (14.8-28.6)	1.0 (0-103)	**2.6** (0.0-100)	19.7 (0.0-19.7)

Table A2.2 Blood phenylalanine values outside the therapeutic range of 120-360 μmol/L, above 360 μmol/L, and below 120 μmol/L among all blood phenylalanine values, per child for children in metabolic control*

Per child, from one month of age to end of follow-up	Children in metabolic control*					
	Classic PKU (n=31)			Less severe PAH deficiency (n=65)		
	Median # (range)	Median % (range)	IQR (25th-75th percentile)	Median # (range)	Median % (range)	IQR (25th-75th percentile)
Values **outside 120-360 μmol/L**	42.0 (2-98)	**31.0** (9.2-39.9)	16.6 (21.3-37.9)	4.0 (0-58)	**10.2** (0-37.5)	20.3 (0.0-20.3)
Values **above 360 μmol/L** [a]	12 (1-64)	**9.7** (1.0-30.0)	18.6 (5.2-23.8)	1 (0-54)	**3.4** (0.0-36.8)	8.3 (0.0-8.3)
Values **below 120 μmol/L** [b]	25 (0-54)	**16.1** (0.0-32.9)	13.1 (9.0-22.1)	0 (0-34)	**0.0** (0-37.5)	9.1 (0.0-9.1)

Table A2.3 Blood phenylalanine values outside the therapeutic range of 120-360 μmol/L, above 360 μmol/L, and below 120 μmol/L among all blood phenylalanine values, per child for children not in metabolic control*

Per child, from one month of age to end of follow-up	Children NOT in metabolic control*					
	Classic PKU (n=49)			Less severe PAH deficiency (n=19)		
	Median # (range)	Median % (range)	IQR (25th-75th percentile)	Median # (range)	Median % (range)	IQR (25th-75th percentile)
Values **outside 120-360 μmol/L**	78 (3-242)	**50** (40.5-88.9)	12.9 (45.5-58.4)	50 (1-133)	**48.5** (40.1-100)	17.1 (42.9-60.0)
Values **above 360 μmol/L** [a]	35 (3-135)	**26.8** (2.6-88.9)	13.5 (17.9-31.4)	14 (0-87)	**20.9** (0.0-60.9)	21.9 (13.9-35.8)
Values **below 120 μmol/L** [b]	44 (0-107)	**24.3** (0-46.5)	10.7 (19.9-30.6)	30 (0-103)	**25.8** (0-100)	14.4 (18.9-33.3)

#: number; Phe: phenylalanine; IQR: Interquartile Range; * A child is considered in metabolic control if more than 60% of their blood phenylalanine values beyond the first month of life are within therapeutic range (i.e., 120-360 μmol/L) [5,25] [a] for each child, we calculated the % as # of phe values >360 μmol/L divided by total # of phe
[b] for each child, we calculated the % as # of phe values <120 μmol/L divided by total # of phe values
